# Efficacy and Safety of Mometasone Furoate Nasal Spray in Treating Nasal Polyposis: A Systematic Review and Meta-Analysis

**DOI:** 10.7759/cureus.70498

**Published:** 2024-09-30

**Authors:** Osamah H Mohya, Alaa A Alghamdi, Alanoud A Alomar, Amal M Alharbi, Amal M Ageeli, Danah H Alharbi, Haya M Alotaibi, Manar S Alghamdi, Marya A Alkhamis, Moath H Shawosh, Renad S Alayidh, Salma A Binmahfoodh, Shahad M Albariqi, Hadi Afandi Al-Hakami, Mohammed Al-Garni

**Affiliations:** 1 Medicine and Surgery, Faculty of Medicine, King Khalid University, Abha, SAU; 2 Medicine and Surgery, Faculty of Medicine, Umm Al-Qura University, Makkah, SAU; 3 Medicine and Surgery, Faculty of Medicine, Princess Nourah bint Abdulrahman University, Riyadh, SAU; 4 Medicine and Surgery, Faculty of Medicine, King Faisal University, Alhasa, SAU; 5 Medicine and Surgery, Faculty of Medicine, Jazan University, Jazan, SAU; 6 Medicine and Surgery, Faculty of Medicine, Taif University, Taif, SAU; 7 Medicine and Surgery, Faculty of Medicine, University of Jeddah, Jeddah, SAU; 8 Medicine and Surgery, Faculty of Medicine, king Faisal University, Alhasa, SAU; 9 Medicine and Surgery, Faculty of Medicine, King Saud University, Riyadh, SAU; 10 Otolaryngology, King Saud bin Abdulaziz University for Health Sciences, King Abdullah International Medical Research Center, Ministry of National Guard Health Affairs, Jeddah, SAU; 11 Otolaryngology-Head and Neck Surgery, King Saud Bin Abdulaziz University for Health Sciences, King Abdullah International Medical Research Center, Riyadh, SAU

**Keywords:** efficacy, mometasone furoate, nasal polyps, randomized controlled trials, safety

## Abstract

Topical and systemic corticosteroids are the most effective medical treatment for nasal polyps, this study aims to systematically explore the efficacy and safety of mometasone furoate (MTF) for patients with nasal polyps (NP). The systematic review was conducted following the preferred reporting items for systematic reviews and meta-analyses (PRISMA) guidelines and was registered in The International Prospective Register of Systematic Reviews (PROSPERO.) Data were extracted from relevant and appropriate randomized controlled trials (RCTs) of synthetic corticosteroids for patients with nasal polyps from PubMed, Google Scholar, Web of Science, and Science Direct, encompassing studies published between January 1995 and July 2024.

A total of 834 publications were found based on the search criteria. Around 1,710 patients were reviewed for this systematic review from full full-text articles. Primary outcomes include a change in polyp size (assessed by total polyp size scores), and nasal congestion (assessed by measuring changes from baseline in nasal congestion/obstruction scores). secondary outcomes consist of nasal symptoms, including loss of smell, anterior rhinorrhea, postnasal drip, quality of life outcomes, and adverse events. Mometasone furoate nasal spray (MFNS) significantly improves nasal symptoms and reduces nasal polyp size for patients with nasal polyps (NP).

## Introduction and background

Nasal polyps are described as painless, soft, and noncancerous lesions on the lining of the nose and paranasal sinuses that occur in groups like grayish grape-shaped growth [1]. Patients with nasal polyposis suffer from nasal congestion and obstruction along with other symptoms such as facial pain, headache, post-nasal drip, and itching around the eyes [2].

 Nasal polyps should be treated because they affect the quality of life by causing a loss of the ability to smell and difficulty breathing, especially during sleep [3]. Medical treatment is the first line for nasal polyposis treatment [4]. Topical and systemic corticosteroids are the most effective medical treatment for nasal polyps, as they effectively reduce polyp size and improve symptoms [5]. Mometasone furoate is a synthetic corticosteroid with a high affinity for glucocorticoid receptors that can be used for the treatment of allergy symptoms such as runny nose, sneezing, and itching [6].

This study aims to evaluate the efficacy and safety of mometasone furoate nasal spray (MFNS) 200µg, administered once daily (QD) or twice daily (BID) compared with placebo.

## Review

Methods and materials

Search Strategy

To guarantee that papers were chosen with the least degree of bias possible, we adhered to the preferred reporting items of systematic reviews and meta-analyses (PRISMA) guidelines in our review [7]. The International Prospective Register of Systematic Reviews (PROSPERO) received this study protocol registration a priori under the following ID: CRD42024572899 [8]. The study's design precluded the need for ethical approval. We performed a thorough search of the following databases in July 2024: (1) PubMed, (2) Google Scholar, (3) Web of Science, and (4) Science Direct for studies published between January 1995 and July 2024. The search only included randomized controlled trials (RCTs). The terms "mometasone furoate or MFNS" and "nasal polyposis or nasal polyps" were used in the search (Appendix 1). Studies were taken into consideration for the review based on population, intervention, comparator, outcome, time, and setting (PICOTS) criteria as follows: adults (18 years old and older) (P), MFNS 200µg (I) compared to placebo (C) changes in bilateral polyp grade and change in nasal congestion/obstruction score (O) over a four-month period (T) in a randomized control trial (S).

Methodology for Selecting Studies

For inclusion in our systematic review, studies had to meet the following criteria: (1) they had to be in the English language, (2) the patients had to be adults (18 years old and older), (3) patients in the study had been diagnosed with bilateral nasal polyps and significant nasal congestion/obstruction, (4) it had to be an RCT (randomized controlled trials) studies, (5) MFNS 200 µg should be administered to patients once daily (QD) or twice daily (BID), (6) studies comparing the MFNS to a placebo nasal spray, (7) primary outcomes should show changes in bilateral polyp grade score and change in nasal congestion/obstruction score, (8) secondary outcomes should include changes in loss of smell, anterior rhinorrhea, postnasal drip scores, peak nasal inspiratory flow (PNIF), and the proportion of subjects showing improvement. Studies were excluded from our systematic review based on the following criteria: (1) patients with a history of seasonal allergic rhinitis, recent nasal or sinus surgery, presumed fibrotic nasal polyposis, complete or near-complete nasal obstruction, or specific clinical conditions (e.g., Churg-Strauss syndrome, cystic fibrosis), (2) studies not involving MFNS or without adequate dosing information, (3) studies did not report any of the aforementioned outcomes of interest, (4) the study was of the following types: Non-randomized trials, observational studies, case reports, or reviews, (5) the study was not in English language.

Process of Screening and Data Extraction

Two independent reviewers screened papers and independently reviewed them by title and abstract using the Rayyan search web and mobile app for systematic reviews [9]. After that, the full texts of the articles were then reviewed by four independent reviewers, and they cross-checked each other’s work. Additionally, any differences are resolved and discussed between them. After which, data extraction was performed by other four reviewers for the following variables: (1) total number of patients who were included, (2) total number of patients in the group who received MFNS once daily with their dosage administered, (3) total number of patients in the group who received MFNS twice daily with the dosage administered, (4) total number of patients in the group who received placebo doses, (5) age range of patients, (6) mean age in years in each group (7) number of patients with male sex, (8) number of patients with female sex, (9) comorbidities, (10) type of intervention, (11) comparison group, (12) dosage, (13) duration of treatment, (14) outcomes being measured (e.g. complications, quality of life, etc), (15) primary outcomes which are change in bilateral polyp grade score and change in nasal congestion/obstruction score, (16) secondary outcomes which are changes in loss of smell, anterior rhinorrhea, postnasal drip scores, peak nasal inspiratory flow (PNIF), and proportion of subjects with improvement. furthermore, Rayyan double-checked the retrieved data to prevent duplication [9].

Assessment of Quality and Bias Risk

RCT papers that we have gathered were assessed for quality using the Cochrane risk of bias tool [10]. Two reviewers documented the total risk of bias for each study, classifying it as low, high, or unclear based on the evaluation of various categories. A third reviewer was consulted to settle any disagreement.

Meta-Analysis of the Included Data

This meta-analysis was conducted using Review Manager 5.4 (The Cochrane Collaboration, London, England, UK). Subgroup analyses were performed to assess heterogeneity between the results of the three studies for both once-daily and twice-daily MFNS treatments. Heterogeneity was measured using the Higgins I² statistic, where I² values were interpreted as follows: I²<25% (assumed homogeneous), I²=25%-50% (low heterogeneity), I²=50%-75% (moderate heterogeneity), and I²>75% (high heterogeneity). A p-value of the Chi-squared statistic was used to assess significance, with p<0.05 indicating significant heterogeneity.

For the selected studies, a random-effects model was applied when the I² value exceeded 50%, indicating moderate or high heterogeneity, particularly in studies involving twice-daily MFNS. Conversely, a fixed-effects model was used when the I² value was less than 50%, which was the case for studies involving once-daily MFNS.

A 95% confidence interval (CI) was calculated to estimate the treatment effects of MFNS compared to control groups across different studies. Statistical significance was set at p<0.05.

Results

The Literature's Findings

**Figure 1:**
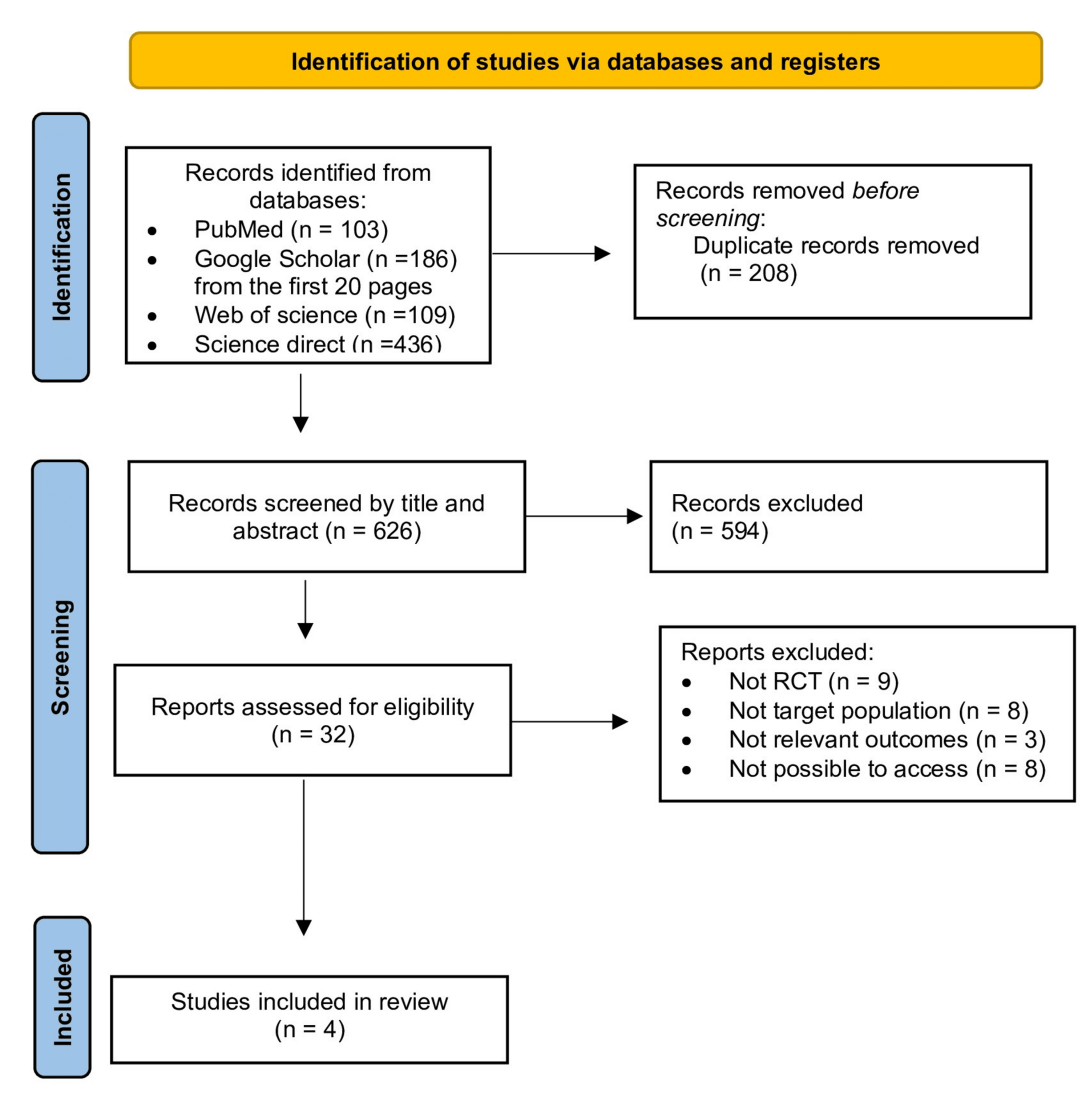
Flow Diagram of Preferred Reporting Items for Systematic Reviews and Meta-Analyses

A comprehensive search of four electronic databases, including PubMed, Google Scholar, Web of Science, and ScienceDirect, yielded a total of 834 records. After removing 208 duplicates, a total of 626 unique records remained. A thorough screening of titles and abstracts led to the exclusion of 594 irrelevant records, resulting in 32 studies for full-text assessment. Of these, 28 studies were excluded due to not being randomized controlled trials (RCTs), not targeting the specified population, lacking relevant outcomes, or being inaccessible. Ultimately, four RCTs meeting all inclusion criteria were included in this review, as illustrated in the PRISMA flow diagram (Figure 1).

A Description of the Characteristics of the Included Studies

All included studies were randomized controlled trials (RCTs) conducted between 2005 and 2015, originating from China, the USA, and Sweden. To minimize bias, these studies employed double-blind or double-dummy blinding techniques, with both participants and investigators unaware of treatment allocation. The study designs were predominantly parallel-group, with a mix of single-center and multicenter settings.

Patient enrollment spanned across single countries like China and the USA, as well as multicenter studies involving countries such as Sweden (including patients from Denmark, Finland, Norway, and Sweden). A total of 1,710 participants with bilateral nasal polyps and clinically significant congestion or obstruction were included in the four studies. Sample sizes ranged from 298 to 748 patients. The studies compared different treatment arms: Group 1 received MFNS at varying doses (n=375, 115, 153, 102); Group 2 received either placebo or MFNS 200 μg twice daily (n=373, 122, 145, 102); and Group 3 served as the placebo control group (n=117, 106). As shown in Table 1.

**Table 1:** Overview of Study Characteristics N: number of patients, QD: once daily, BID: twice daily, MFNS: mometasone furoate nasal spray, RCT: randomized controlled trial, N/A: not applicable.

Study ID	Journal	Study design	Year of publication	Country of origin	N	N interventional group (MFNS 200 mcg QD)	N interventional group (MFNS 200 mcg BID)	N comparison group (placebo)
Small et al. [11]	The Journal of allergy and clinical immunology	RCT	2005	USA	354	115	122	117
Stjärne et al. [12]	Acta Oto-Laryngologica	RCT	2006	Sweden (Patients were enrolled across centers in Denmark, Finland, Norway, and Sweden).	298	153	N/A	145
Stjärne et al. [13]	Arch Otolaryngol Head Neck Surg.	RCT	2006	Sweden	310	102	102	106
Zhou et al. [14]	International Forum of Allergy and Rhinology	RCT	2015	China	748	N/A	375	373

Primary outcomes across studies focused on changes in nasal congestion/obstruction and polyp size. These were assessed by measuring changes from baseline in nasal congestion/obstruction scores and total polyp size scores at four months. One study additionally evaluated the proportion of participants experiencing improvement in both nasal congestion and polyp size. Secondary outcomes encompassed a broader range of nasal symptoms, including loss of smell, anterior rhinorrhea, postnasal drip, and peak nasal inspiratory flow. Additionally, studies assessed therapeutic improvement, quality of life, and safety outcomes such as adverse events, with epistaxis being the most commonly reported treatment-related adverse event. Participants across the studies were predominantly adults aged 18 to 86 years, with a mean age ranging from 46.7 to 53 years. The majority of participants were male (59.7% to 74.5%), with a relatively equal distribution between treatment groups. Asthma was a common comorbidity reported in the included studies, with prevalence ranging from 2.7% to 19%. Additionally, other allergic conditions such as perennial allergic rhinitis were present in a significant proportion of patients, with rates varying from 14% to 22%. All included studies compared MFNS to placebo. Dosing regimens varied across studies, with MFNS administered once daily (200 µg) or twice daily (200 µg). Placebo sprays were matched to the active treatment in terms of appearance and administration. The treatment duration for all studies was four months.

Patient-Reported Outcomes, Complications, and Clinical Outcomes

Across four studies, MFNS demonstrated consistent efficacy in treating various nasal symptoms associated with nasal polyposis. Administered once or twice daily at a dose of 200 µg, MFNS significantly reduced polyp size (mean difference range: -0.76 to -1.15), improved nasal congestion (mean difference range: -0.86 to -1.10), alleviated anterior rhinorrhea (mean difference range: -0.53 to -0.74), postnasal drip (mean difference range: -0.37 to -0.62), and loss of smell (mean difference range: -0.33 to -0.60) compared to placebo. Additionally, MFNS showed improvements in nasal patency, as measured by Peak nasal inspiratory flow (PNIF) scores, with a mean difference range of 36.1 to 47.1. These findings support MFNS as an effective medical treatment for nasal polyposis, potentially reducing or delaying the need for surgical intervention. The medication was well-tolerated across study populations, with improvements in nasal congestion, polyp size, and other symptoms.

Meta-Analysis of Once-Daily MFNS

**Figure 2:**
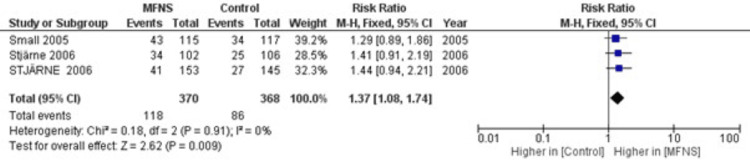
Forest Plot of the Risk Ratio for Subjects With an Improvement With MFNS Once Daily Compared to Placebo [11-13], MFNS: mometasone furoate nasal spray.

A fixed-effects meta-analysis of three studies involving 368 control participants and 370 participants treated with once-daily MFNS demonstrated a significantly increased proportion of subjects with improvement in nasal congestion, polyp size, and other symptoms in the MFNS group compared to the control group [risk ratio (RR)=1.37, 95% confidence interval (CI): 1.08, 1.74, p<0.009]. The choice of a fixed-effects model was justified by the minimal heterogeneity observed among the studies, as evidenced by a low I² statistic (0%) and a non-significant chi-square test for heterogeneity (Chi²=0.18, df=2, p=0.91). These findings are visually represented in the forest plot in Figure 2.

Meta-Analysis of Twice-Daily MFNS

**Figure 3:**
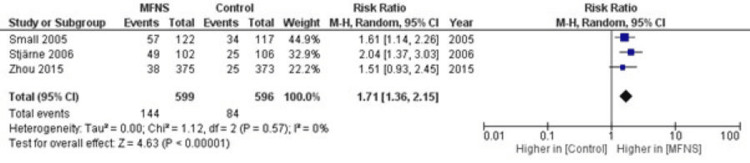
Forest Plot of the Risk Ratio for Subjects With an Improvement With MFNS Twice Daily Compared to Placebo [11,13,14], MFNS: mometasone furoate nasal spray.

A random-effects meta-analysis was conducted to assess the efficacy of twice-daily MFNS. This approach was chosen after evaluating heterogeneity using a fixed-effects model, which revealed significant heterogeneity. However, when a random-effects model was employed, minimal heterogeneity was observed (I²=0%, p=0.57, Chi²=1.12, df=2), suggesting consistent results across the studies. The analysis included three studies involving 596 control participants and 599 participants treated with twice-daily MFNS. The results demonstrated a significantly increased proportion of subjects with improvement in nasal congestion, polyp size, and other symptoms in the MFNS group compared to the control group (RR=1.71, 95% CI: 1.36, 2.15, p<0.00001). These findings are visually represented in the forest plot in Figure 3.

Analyzing Biases, Assessing Quality, and Determining the Level of Evidence

The risk of bias was assessed using the Cochrane Risk of Bias tool (RoB 2) (Table 2). Two studies were deemed high risk, while two others were low risk. This heterogeneity in study quality limits the strength of the evidence. However, a sensitivity analysis excluding high-risk studies did not substantially alter the main findings, suggesting that the observed effect is robust.

**Table 2:** Cochrane Risk-of-Bias Tool for Randomized Trials (RoB 2) RCT: randomized controlled trial, RoB: risk of bias, H: high, L: low, N/A: not applicable.

Study ID	Study Type	Risk of Bias Tool	Selection Bias	Performance Bias	Detection Bias	Attrition Bias	Reporting Bias	Other Bias	Overall RoB
Small et al. [11]	RCT	Cochrane RoB	H	L	N/A	L	H	None	H
Stjärne et al. [12]	RCT	Cochrane RoB	L	L	L	L	L	None	L
Stjärne et al. [13]	RCT	Cochrane RoB	L	L	L	L	L	None	L
Zhou et al. [14]	RCT	Cochrane RoB	L	L	N/A	L	L	None	H

Discussion

For individuals with chronic rhinosinusitis with nasal polyps, the topical use of intranasal corticosteroids (INCSs) has been a common method of managing illness symptoms. A recent systematic analysis examining the impact of INCSs indicated that patients' symptoms significantly improved [15]. The objective of our systematic review was to compare the effectiveness and safety of MFNS 200 µg with a placebo when it was taken once daily (QD) or twice daily (BID). The results of our review indicate that MFNS administered once or twice daily at a dose of 200µg is effective in reducing polyp size, improving nasal congestion, alleviating anterior rhinorrhea, relieving postnasal drip, improving the sense of smell and nasal potency. These results suggest that the use of MFNS can postpone or lessen the necessity for surgery.

Our results align with earlier research on the subject. A study by Joe et al. It was a systematic review study with meta-analysis aimed to compare different types of intranasal steroids (INS) to determine their usefulness in the management of chronic rhinosinusitis without polyps and chronic rhinosinusitis (CRS) with polyps, and according to the results of their analysis, mometasone one of the intranasal steroids which is effective for the treatment of sinonasal polyposis (SNP) in adults [16]. Our review also revealed that Mometasone furoate can alleviate anterior rhinorrhea, which is consistent with the findings of Fandiño et al. They conducted a systematic review with meta-analysis, and they gathered studies with different types of intranasal corticosteroids to evaluate the effectiveness of INCSs for patients requiring functional endoscopic sinus surgery in the postoperative period, and the only study that used mometasone furoate as their intervention showed significant improvement in reducing rhinorrhea [17]. From a clinical practice implication view, the use of intranasal corticosteroids as postoperative care of patients with chronic rhinosinusitis with nasal polyps after endoscopic surgery suggests a reduction in the recurrence of polyps and shows improvement in polyp score and symptom scores (derived from outdated low-to-moderate-quality evidence) [17]. All the studies included in our systematic review were randomized controlled trials, double-blinded, placebo-controlled studies. This is an important point whenever testing a particular drug as randomized control trials are the best and gold standard method to test the efficacy and safety of the drug. A noteworthy aspect is that the duration of treatment of MFNS in all the studies included was four months and it is sufficient as regards that reducing the size of nasal polyps is thought to be a slow process, therefore, to achieve the primary and secondary outcomes, the patient should take MFNS regularly and constantly [11]. Also, the included studies had an appropriate sample size and were all conducted in different centers and countries to ensure that the efficacy, safety, and adverse events of MFNS are well documented and good enough to be safely administered to the patients. MFNS is well-tolerated and has proven its efficacy in relieving nasal symptoms and reducing the size of nasal polyps among the patients included in the studies. However, one limitation in the included studies is the sample size of the patients despite being 18 years old and older, none of them mention or highlight the effects of MFNS in pregnant women. As a result, We recommend more Studies to be conducted about the efficacy and safety of mometasone furoate nasal spray on pregnant women, as nasal obstruction, post-nasal drip, nasal itching, and nasal discharge are common symptoms during pregnancy [18], which can impact the quality of life of the pregnant woman from the aspect which could result in obstructive sleep apnea, daytime tiredness and emotional changes of a pregnant woman which may harm the fetus [19]. Another noteworthy point mentioned by Stjärne et al. is that using the medication bottle weight as a means of measuring compliance is prone to manipulation by nonadherent patients to improve their reported compliance [13]. We recommend future studies to involve the technology as possible to monitor the patient’s adherence to the medication, as the non-adherence to the medication is a concern for treatment failure especially in the medications that require full course and long-term treatment [20]. For instance, inhaler attachments which contain a computer chip that records dates and times of device manipulations (e.g., inhaler actuations, bottle/box openings) can give us accurate data and allow us to monitor the patient’s adherence to the medication [21]. However, This systematic review and meta-analysis included only a relatively small number of studies despite our efforts to gather data from numerous databases. This was due to our strict inclusion and exclusion criteria, which excluded studies involving patients with conditions such as seasonal allergic rhinitis, recent nasal or sinus surgery, fibrotic nasal polyposis, or specific clinical conditions (e.g., Churg-Strauss syndrome, cystic fibrosis). Additionally, studies were excluded if they did not involve MFNS with adequate dosing or did not report the outcomes of interest. As a result, the pool of eligible RCTs was reduced, limiting the sample size of the meta-analysis.

## Conclusions

We performed a high-quality systematic review and meta-analysis of studies assessing the efficacy and safety of mometasone furoate nasal spray in treating nasal polyposis. The studies showed that using mometasone furoate nasal spray once or twice daily produced significantly greater reductions in bilateral polyps. It was also safe and significantly superior to placebo in reducing polyp grade (size and extent), improving congestion/obstruction, and returning a sense of smell. However, despite our extensive efforts to compile data from multiple databases, the number of studies included in our systematic review and meta-analysis remained limited. This is because only a small number of randomized controlled trials met the strict inclusion and exclusion criteria established for this analysis. We suggest further studies be done, including randomized control trials with a longer follow-up duration. Appropriate patient selection is important in achieving excellent outcomes.

## Appendices

Appendix 1 

**Table 3:** Search Strategy MFNS: mometasone furoate nasal spray.

Database	Search strategy (medical subject headings (MeSH) terms or medical keywords/synonyms used)
PubMed	("mometasone furoate"[MeSH Terms] OR "MFNS" OR "mometasone furoate") AND ("nasal polyposis"[MeSH Terms] OR "nasal polyps" OR "nasal polyposis")
Google scholar	"mometasone furoate" OR MFNS AND "nasal polyps" OR "nasal polyposis"
Web of science	TS=("mometasone furoate" OR "MFNS") AND TS=("nasal polyps" OR "nasal polyposis")
Science direct	("mometasone furoate" OR MFNS) AND ("nasal polyps" OR "nasal polyposis")
